# Acceptability, Feasibility, and Perceived Usefulness of the School eHealth Education Program Pakistan (eSHEPP) for Improving Adolescents’ Awareness of Noncommunicable Diseases in Secondary and Higher Secondary Schools

**DOI:** 10.21203/rs.3.rs-8270459/v1

**Published:** 2025-12-15

**Authors:** Muhammad Shahid Khan, Aysha Almas, Zainab Samad, Kanecia Obie Zimmerman, Tazeen Saeed Ali

**Affiliations:** Aga Khan University; Aga Khan University; Aga Khan University; Duke University; Aga Khan University

**Keywords:** Noncommunicable diseases (NCDs), adolescent health, eHealth, school-based intervention, digital literacy, Pakistan, qualitative research

## Abstract

**Background:**

Noncommunicable diseases (NCDs) are a leading cause of premature morbidity and mortality, and many risk behaviors emerge during adolescence. In Pakistan, school health education remains limited and primarily didactic, leaving adolescents insufficiently equipped to adopt healthy behaviors. To address this gap, the School eHealth Education Program Pakistan (eSHEPP), a multimedia, app-supported intervention, was developed. This study aimed to explore secondary and higher secondary students’ perceptions of eSHEPP’s acceptability, feasibility, and perceived usefulness in increasing awareness of NCDs following program delivery in school settings.

**Methods:**

A qualitative explanatory study, guided by an integrated Technology Acceptance Model (TAM) and Task–Technology Fit (TTF) framework, was conducted in four schools (two secondary, two higher secondary; two all-girls, two all-boys) in Karachi. eSHEPP was delivered over eight weeks through six classroom-based sessions. Each 20–30-minute session included a 7–10-minute dramatized video, followed by guided discussion and interactive Q&A led by a trained facilitator and teacher representative. A total of 27 students participated in four focus group discussions (FGDs) conducted after program completion. Data were collected using piloted semi-structured guides in Urdu and analyzed thematically using a hybrid deductive–inductive approach in NVivo, following COREQ guidelines.

**Results:**

Students described eSHEPP as highly acceptable and user-friendly, appreciating the dramatized videos, clear language, and supportive subtitles. Most participants reported increased awareness of NCDs and risk factors, healthier dietary choices, greater recognition of the importance of physical activity, and peer-led discouragement of smoking. The program’s smooth integration into school schedules and minimal resource demands (technological and personnel) enhanced perceived feasibility. Participants suggested expanding health topics, slightly extending session duration, and incorporating interactive features such as quizzes, games, and peer-engagement tools to sustain interest.

**Conclusions:**

eSHEPP demonstrated strong acceptability, high feasibility, and clear educational value in under-resourced Pakistani schools. With refinements in interactivity and delivery, and formal integration into school curricula supported by teachers and parents, eSHEPP has the potential to be a scalable and sustainable model for adolescent health promotion in low-resource settings. Future studies should evaluate long-term outcomes using mixed-method and longitudinal designs.

## BACKGROUND

Adolescents constitute a rapidly expanding population in low- and middle-income countries (LMICs), and their health behaviors are likely to shape the trajectory of the noncommunicable disease (NCD) epidemic. NCDs account for the majority of global deaths, with LMICs disproportionately affected by premature mortality (1–3). A substantial proportion of premature adult deaths can be traced to risk behaviors established during childhood and adolescence, including physical inactivity, unhealthy diet, tobacco and substance use, and overweight or obesity (4, 5). The World Health Organization (WHO) has identified the rising prevalence of NCD risk factors among adolescents as a critical global health concern (6). Adolescence represents a window of opportunity for establishing lifelong healthy behaviors, as young people navigate complex physical, psychological, and social transitions (7). WHO estimates indicate that NCDs cause 74% of global deaths, with 82% of premature NCD deaths occurring in LMICs, underscoring adolescence as a formative period for future health trajectories (4, 8, 9).

Health education is fundamental to NCD prevention. Evidence suggests that adolescents require both knowledge and practical skills to make informed health decisions, with interactive, school-centered programs consistently outperforming traditional didactic approaches (10). Strategies such as model-driven trials and participatory techniques are essential for enhancing both short- and long-term health outcomes (11). In line with WHO’s Health-Promoting Schools framework, well-designed, student-centered programs can foster healthier behaviors; however, many education systems in LMICs remain under-resourced and inconsistently implemented (12, 13).

In Pakistan, structured school health programs are scarce, and evidence on effective delivery strategies is limited. National analyses have described school health as an “ignored domain”, emphasizing the need for scalable, context-sensitive interventions (14). Digital technologies offer innovative, accessible, and cost-effective solutions to enhance student health literacy. They can promote healthy eating, physical activity, and chronic disease prevention, while leveraging mobile connectivity, social media, and gamified learning to engage adolescents (15, 16). Evidence shows that digital and mass media campaigns can positively shape health norms and behaviors (8, 17), and adolescents are increasingly accessing mobile apps, peer networks, text-based health programs, and online mental health resources (18–20). eHealth interventions have demonstrated potential to improve adolescent health outcomes and reduce health inequities, particularly in LMICs such as Pakistan (8, 15, 19–24). This opportunity is significant in Pakistan, where nearly one in five people is aged 10–19 years, and mobile connectivity is widespread (≈ 198 million cellular subscribers and > 134 million mobile broadband users as of June 2025). However, digital divides persist, especially by gender and digital literacy (25–27). Systematic reviews highlight that well-designed eHealth and mHealth interventions can enhance physical activity, mental health, and self-management of NCDs when usability and engagement are prioritized (28–30).

Despite growing recognition of adolescent health needs, school-based health education in Pakistan remains fragmented and underdeveloped, often lacking interactive or participatory methods (31). Health topics are rarely integrated into core curricula, and public-sector schools face constraints including limited infrastructure, trained staff, and cultural sensitivities around health discussions (32). Consequently, students remain underprepared to make informed health choices. Early evidence suggests that digital, school-based lifestyle programs are feasible and scalable, though acceptability and contextual fit remain key determinants of success (33).

With the rapid rise in mobile phone usage across Pakistan (34), digital health platforms present an opportunity to deliver scalable, engaging, and culturally relevant health education to adolescents (15, 16). However, evidence remains limited regarding students’ perceptions, acceptability, and feasibility of integrating digital health education within school systems. To address this gap, the School eHealth Education Program Pakistan (eSHEPP) was developed as a digital health education initiative targeting secondary and higher secondary students. The program delivers culturally sensitive, multimedia content via videos, a mobile app, and classroom sessions focused on major NCD risk factors, such as unhealthy diet, physical inactivity, mental well-being, tobacco use, and substance abuse. This study aimed to examine secondary and higher secondary school students’ perceptions of the usefulness, acceptability, and feasibility of eSHEPP in improving adolescents’ awareness of NCDs.

## METHODS

### Study design

This study employed a qualitative explanatory design with a descriptive approach, which is well suited to examining participants’ perspectives and lived experiences. The explanatory component provided deeper understanding of how and why students interacted with eSHEPP’s digital content, app features, and health messages.

#### Theoretical framework

**Figure 1:**
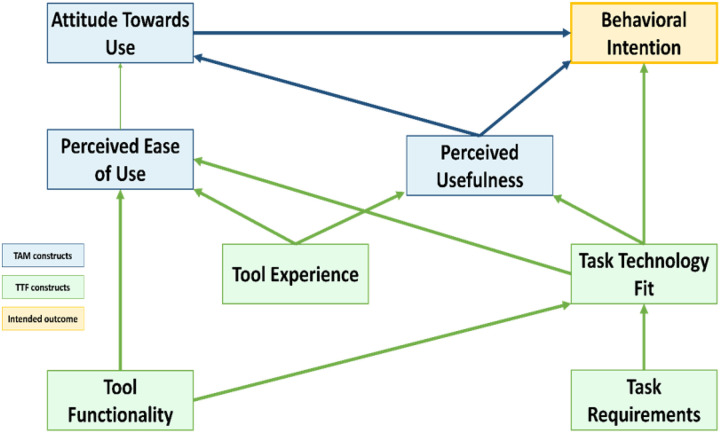
Integrated model of the Technology Acceptance Model (TAM) and Task–Technology Fit (TTF), adapted from Shih and Chen (2013)

The study was guided by an integrated framework combining the Technology Acceptance Model (TAM) and the Task–Technology Fit (TTF) model (Fig. 1), adapted from Shih and Chen (2013) (35). TAM constructs (perceived usefulness, perceived ease of use, and intention to use) informed questions about students’ views on the relevance and usability of eSHEPP, while TTF constructs (alignment with learning needs, task–technology fit, and perceived impact on performance) shaped questions about whether the program supported students in applying health knowledge.

The integrated model posits that perceived usefulness and ease of use influence task–technology fit, which in turn affects engagement and learning performance. This framework also guided a hybrid coding strategy, combining deductive codes drawn from TAM–TTF with inductive themes emerging from student narratives. A detailed codebook, including all themes, subthemes, definitions, and mapped TAM/TTF constructs, is provided in Supplementary Material 5.

#### Sampling technique and participants

The study was conducted in public secondary and higher secondary schools in District East and District Central of Karachi, Pakistan. These districts were included after formal permission from the Directorate of School Education (DSE), Karachi, which authorized and directed the implementation of the eSHEPP program. District selection was therefore determined administratively by the DSE rather than by the research team.

Following DSE approval, the respective District Education Offices (DEOs) were instructed to identify suitable schools for program implementation. In coordination with school administrators, each DEO selected four schools, one boys’ and one girls’ school per level (secondary and higher secondary), resulting in a total of eight schools designated for participation. The research team was not involved in the school selection process.

The eight schools were grouped into four clusters, and randomization assigned four schools to the intervention group and four to the control group. Details of the pilot cluster randomized controlled trial, part of this larger mixed-methods study described in the published protocol (36).

The current qualitative phase was conducted in the four intervention schools to evaluate the feasibility, acceptability, and usefulness of implementing the eSHEPP program in real-world school settings. Although the original protocol proposed two focus group discussions (FGDs), four FGDs were ultimately conducted to ensure broader gender representation and variation across participating schools. A total of 27 students participated, including 12 males and 15 females, who were selected using a purposive sampling technique to capture diverse perspectives.

#### Intervention delivery

The School eHealth Education Program Pakistan (eSHEPP) is a digital, school-based health education intervention designed to improve adolescents’ awareness of noncommunicable diseases (NCDs) and promote healthy behaviors. The intervention was implemented according to a previously published mixed-methods protocol (36). Development of the intervention was informed by an earlier qualitative exploration phase, which identified barriers and facilitators to implementing digital health education in secondary and higher secondary schools in Karachi and examined stakeholders’ views on the content and design of the proposed eSHEPP program (37). The insights generated during that phase supported refinement of the educational videos, clarified the structure of session delivery, and guided improvements to the digital platform used in the current implementation.

The program was delivered through a custom-built eHealth application accessible on both mobile devices and web browsers, ensuring accessibility for students with varying levels of technological access. The app hosted Urdu-language educational videos, interactive quizzes, and pre- and post-assessments, enabling students to review content and monitor progress. It was hosted on a secure, university-managed server with restricted access for authorized users.

The eSHEPP curriculum comprised six structured multimedia sessions delivered over eight weeks during regular classroom hours. Each session lasted approximately 20–30 minutes and followed a standardized format featuring a brief educational video, a facilitated group discussion, and an interactive question-and-answer segment co-led by a trained facilitator and a teacher representative. The videos used dramatized storytelling and animation to enhance engagement, following an adolescent protagonist who encouraged his family to adopt healthier habits, such as improving diet, increasing physical activity, and avoiding smoking and substance use. The core health topics were understanding NCDs, healthy eating (Eat Smart), physical activity (Keep Moving), avoiding smoking and drugs (Run Away from Smoking and Drugs), and overall wellness (Stay Well).

After each classroom session, students accessed the eSHEPP app individually to review session materials, complete Q&As, and reinforce learning. The app provided simple navigation, self-paced sessions, and instant feedback. All sessions were held during regular school hours to promote inclusivity and minimize academic disruption. Facilitators received standardized training, presented the app through multimedia, played the session videos, addressed student questions during discussions, and recorded attendance, following up with absent students to provide missed content. They also completed post-session fidelity checklists to ensure consistent delivery across schools.

The feasibility and potential efficacy of eSHEPP in school settings were established in a pilot cluster randomized controlled trial, the full results of which will be reported in a separate manuscript. These findings provide context for the current qualitative study, which explores students’ experiences, acceptability, and engagement with the program delivered using standardized procedures, including facilitator training and post-session fidelity checks.

#### Data collection

A semi-structured FGD guide was developed based on the TAM and TTF constructs and aligned with eSHEPP’s digital content. The guide was translated into Urdu and piloted for clarity, cultural relevance, and contextual fit. Following the pilot, no major modifications were required; only minor adjustments were made to the Urdu version to enhance clarity and ease of understanding. The complete guide is provided in Supplementary Material 6.

Focus group discussions were conducted after students completed all six eSHEPP sessions to explore their views on usefulness, acceptability, feasibility, and usability of both the mobile and web versions. Discussions examined engagement with the dramatized videos and animations, interpretation of the storyline, and application of lessons to everyday life.

Each FGD lasted 30–45 minutes and was conducted in school activity rooms to ensure privacy and comfort. Sessions were moderated by a trained qualitative researcher (not affiliated with the schools) and supported by a note-taker. Gender-matched facilitators were included where feasible to enhance comfort and participation. Written parental consent and student assent were obtained. All FGDs were audio-recorded, transcribed verbatim, and verified within two weeks. Data collection continued until thematic saturation was reached, defined as the point at which no new codes or concepts emerged.

### Data analysis

A hybrid thematic analysis was conducted, combining deductive codes derived from TAM–TTF constructs with inductive codes emerging from participant narratives. This allowed exploration of both theoretical domains (perceived usefulness, ease of use, and task–technology fit) and contextual insights related to students’ lived experiences.

Transcripts were managed using NVivo (version 10). Two trained researchers independently coded an initial subset to develop a preliminary coding framework, which was refined iteratively through consensus discussions.

Particular attention was given to students’ experiences with the mobile and web app versions, their interaction with video-based storytelling, and how eSHEPP influenced awareness and behavior, consistent with TAM–TTF constructs. Gender and school differences were also examined. Illustrative quotations were selected to represent diverse perspectives. The analytic process adhered to Consolidated Criteria for Reporting Qualitative Research (COREQ) guidelines to ensure credibility, dependability, confirmability, and transferability (see Supplementary Material 4 for the COREQ checklist).

#### Trustworthiness and rigor

To maintain rigor, the study followed Lincoln and Guba’s four criteria (38). Credibility was enhanced through prolonged participant engagement, gender-matched facilitation, and team debriefings. Member checking was conducted by sharing summarized findings with participating students, who confirmed that the interpretations accurately reflected their perspectives. No further suggestions were provided, and only minor refinements were made to the thematic descriptions.

Dependability was supported through transparent documentation of all methodological steps and maintenance of an audit trail in NVivo and research logs. Confirmability was ensured through reflexive journaling by the lead qualitative researcher and regular peer debriefings to reduce bias. Transferability was promoted through detailed contextual descriptions of the study setting, participant characteristics, and eSHEPP implementation.

#### Researcher reflexivity

FGDs were moderated by a male qualitative researcher with a public health background, fluent in English and Urdu, and experienced in community-based qualitative research. He had no prior relationship with the schools or students, minimizing response bias.

To enhance comfort, gender-matched co-facilitators were included where possible. The researcher maintained a reflexive journal to record assumptions, methodological decisions, and reflections. Regular team debriefings after each FGD and during analysis ensured interpretive balance and analytic transparency.

### Ethical considerations

Ethical approval was obtained from the Aga Khan University Ethical Review Committee (Ref No. 2023–9277-27367). Written informed consent from parents and assent from students were obtained. Data were stored on password-protected systems, and audio recordings were destroyed following transcription and verification. Any modifications to the protocol required approval from the project steering committee and ethics committee.

## RESULTS

After two months of delivering eSHEPP in four schools (two secondary and two higher secondary; two all-girls and two all-boys), a total of 27 students who attended all six sessions participated in four FGDs. The FGDs included two groups with male students (n = 12) and two with female students (n = 15) to capture gender-specific perspectives. All participants had completed six classroom-based, multimedia health-promotion sessions facilitated by a trained facilitator and a teacher representative using the eHealth app. Participant demographics are summarized in Supplementary Material 1.

**Figure 2:**
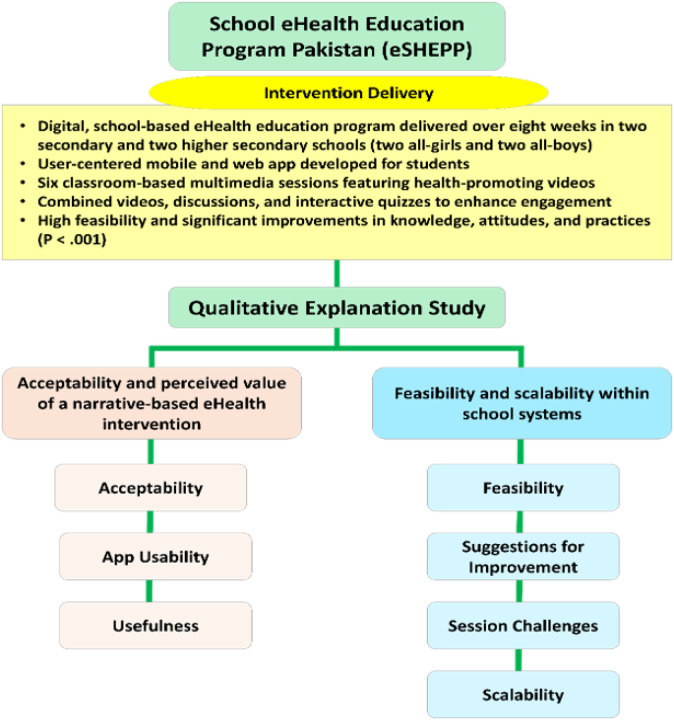
Conceptual framework illustrating intervention delivery and key qualitative themes and subthemes of eSHEPP

Thematic analysis identified two overarching themes encompassing seven interrelated subthemes, as depicted in Fig. 2. The first overarching theme, Acceptability and perceived value of a narrative-based eHealth intervention, comprised the subthemes Acceptability, App Usability, and Usefulness. The second theme, Feasibility and scalability within school systems, included the subthemes Feasibility, Suggestions for Improvement, Session Challenges, and Scalability (see Supplementary Material 3 for the detailed coding tree). Overall, students reported high acceptability and perceived usefulness of the intervention, minimal disruption to academic routines, and positive behavioral spillovers, such as healthier eating, discouraging peers from smoking, and sharing health information with family members.

### Acceptability and perceived value of a narrative-based eHealth intervention

#### Acceptability

Acceptability captured students’ overall reception and engagement with eSHEPP, reflecting their enjoyment, attention, and interaction with facilitators (TAM: Perceived Usefulness & Ease of Use). Students highlighted the value of multimedia, interactive learning, and supportive facilitation as major strengths. Sessions were also seen as a refreshing break from traditional classroom routines.

Students highlighted that visuals, storytelling, and dramatization in videos made health concepts memorable and easy to share at home.

“It was interesting; I enjoyed it.”

(FGD1, Female)

“The information… was very helpful. We shared this with our family members.”

(FGD3, Male)

The novelty of video-based learning sustained attention and motivated discussions beyond the classroom.

“It was something different from regular studies, making it more interesting.”

(FGD1, Female)

“We made lifestyle changes, and we shared this with our family members, so many changes occurred in our lifestyle.”

(FGD3, Male)

Students appreciated facilitators who clarified doubts and encouraged interaction, creating a supportive environment.

“They helped clarify all our doubts.”

(FGD1, Female)

“Whatever was discussed was explained to us, and if we didn’t understand anything, the staff provided further explanation.”

(FGD3, Male)

“The atmosphere was very friendly.”

(FGD2, Female)

Students rated sessions highly (9–10/10), emphasizing that they were both enjoyable and educational.

“The program was quite good. It was enjoyable… and it felt good. It refreshed our minds.”

(FGD1, Female)

“I’d rate it 10 out of 10 because it was engaging and helpful.”

(FGD2, Female)

#### App usability

App usability examines whether the eHealth app interface supported effective learning (TAM: Perceived Ease of Use). Students found the app intuitive, visually clear, and easy to navigate, which enhanced engagement and allowed them to focus on content rather than technical aspects. Simple design, clear visuals, and functional controls contributed to a positive user experience.

The app was easy to navigate, with intuitive controls, making learning accessible and engaging for students.

“I would recommend it; the app is very user-friendly and easy to navigate.”

(FGD2, Female)

“Everything was simple and easy to understand.”

(FGD3, Male)

“I would recommend this app to others because it’s genuinely helpful for learning.”

(FGD2, Female)

Clear, uncluttered interface with no confusing features, supporting smooth interaction and comprehension.

“The app was straightforward, no confusing steps or complicated features.”

(FGD3, Male)

“No issues at all; everything was clear and functional.”

(FGD1, Female)

#### Usefulness

Usefulness measures whether students perceived tangible benefits from the program in terms of knowledge, attitudes, and behavior (TAM: Perceived Usefulness). Students reported substantial learning gains about noncommunicable diseases (NCDs), healthy lifestyles, and mental health. They also described behavior changes, both personally and in influencing peers and family members.

Students reported substantial learning gains about noncommunicable diseases (NCDs), healthy lifestyles, and mental health. Many described this as a meaningful opportunity to learn topics that were previously unfamiliar or confusing.

“The program taught us many new things; topics we didn’t understand before are now clear.”

(FGD1, Female)

“We learned so much through this program, especially about how smoking, physical activity, and diseases are connected.”

(FGD1, Female)

“Before the sessions, we knew very little. But after watching the videos and hearing explanations, we learned so much more.”

(FGD3, Male)

Students also described positive changes in attitudes, noting that the sessions helped them reflect critically on their own health behaviors and choices.

“Before these sessions, we ate oily foods without realizing they could lead to health problems. Now we understand the risks much better.”

(FGD1, Female)

“I understood walking was healthy but didn’t realize it qualified as proper physical exercise until these sessions.”

(FGD2, Female)

“We learned it’s important to share our feelings with others; this helps reduce stress and prevents mental health issues.”

(FGD1, Female)

Several participants further described changes in their practices and everyday habits, showing that lessons learned translated into tangible actions at home and with peers.

“After learning about diabetes, I advised my mother to cut back on sugar, and she actually listened!”

(FGD3, Male)

“When I saw my friend smoking, I intervened and invited him to learn more about the risks.”

(FGD4, Male)

Overall, students viewed the sessions as highly beneficial, emphasizing that they were both enjoyable and educational.

“The program was truly valuable; I learned a lot and enjoyed the sessions.”

(FGD2, Female)

“I would rate the program benefit as a 9 out of 10; it was highly valuable.”

(FGD4, Male)

### Feasibility and scalability within school systems

#### Feasibility

Feasibility assessed how easily eSHEPP could be integrated into school schedules, considering practical constraints and resources (TTF: Task–Technology Alignment). Students highlighted that sessions were practical, resource-light, and flexible, allowing delivery without disruption to academic activities. Simple video discussions could be conducted in any classroom, and teacher involvement enhanced focus and participation.

Sessions fit smoothly into the school timetable, providing structured breaks similar to physical education or other enrichment periods.

“The sessions were like PT (Physical Training) periods, enjoyable but still structured, so they didn’t disrupt our regular studies.”

(FGD2, Female)

“Just like we have a dedicated sports period, we could also have regular sessions like this.”

(FGD3, Male)

The program required minimal resources and could be delivered in diverse classroom settings.

“We don’t need expensive equipment or complicated setups, just basics like a laptop and speaker.”

(FGD3, Male)

“This program works anywhere, no special setup needed.”

(FGD2, Female)

Teacher presence increased engagement, discipline, and students’ perception of the program’s importance.

“Having the teacher present kept everyone focused, no one disrupted the session.”

(FGD4, Male)

“When teachers are involved, it makes us feel the program matters - we take it more seriously.”

(FGD2, Female)

Weekly sessions were preferred, balancing retention of knowledge with sustained interest.

“Weekly sessions help us remember better without losing interest.”

(FGD3, Male)

“Daily sessions could make it feel repetitive and less exciting.”

(FGD2, Female)

Videos were clear and concise; dramatization and subtitles improved comprehension. Most students preferred 7–10 minutes, though some suggested slightly longer sessions for deeper learning.

“Absolutely, the drama made everything clear, leaving no confusion.”

(FGD1, Female)

“The English subtitles were very helpful; they kept us engaged and made it easier to follow along.”

(FGD3, Male)

“The video length felt normal; not too short or too long.”

(FGD1, Female)

“A 20-minute session worked well, it’s short enough not to disrupt our schedule.”

(FGD4, Male)

“Slightly longer videos would make it more enjoyable.”

(FGD1, Female)

### Suggestions for improvement

Students provided thoughtful recommendations to enhance engagement and effectiveness, reflecting a strong sense of ownership and investment in eSHEPP (TAM/TTF: Refinement of Fit). Many students suggested expanding the program’s content beyond the current focus on a single disease to include a wider range of health issues.

“We want more content; currently, it only covers one disease. Expanding to other health issues would be valuable.”

(FGD4, Male)

“We need more stories like Kamran’s journey; one example is good, but more would be better.”

(FGD1, Female)

“Including Islamic practices like Namaz in the videos would be beneficial; it’s both spiritually meaningful and physically active.”

(FGD2, Female)

“Before-and-after photos of smokers would powerfully demonstrate the effects.”

(FGD3, Male)

Several participants also proposed increasing the duration of sessions slightly to allow for deeper discussions and enhanced engagement.

“Extending the session length slightly could enhance engagement and make them more enjoyable.”

(FGD1, Female)

“Slightly longer sessions would make the experience more engaging and enjoyable.”

(FGD2, Female)

Students further emphasized the value of interactive digital features to increase user participation and enjoyment. Suggestions included adding voice recording options, incorporating health-related games, and enabling features for peer interaction and content sharing.

“The app should include a voice recording feature; this would let students explain concepts more clearly.”

(FGD3, Male)

“Adding interactive games to the app would help us learn concepts more effectively.”

(FGD3, Male)

“The app should allow friend chats to easily share helpful videos.”

(FGD4, Male)

“Adding playback controls like fast-forward or full-speed options would help us review content more efficiently.”

(FGD1, Female)

### Session challenges

Students also identified barriers and constraints encountered during eSHEPP sessions (TTF: Constraints on Fit). These included technical difficulties, background noise, and classroom seating arrangements that occasionally hindered engagement and comprehension. Addressing these constraints is important for ensuring inclusivity and maximizing program effectiveness.

“The poor video and audio quality made it difficult to understand, especially with all the background noise.”

(FGD4, Male)

“When other students talked during sessions, we missed key information.”

(FGD4, Male)

“Older students occupied the front rows, leaving younger students in the back struggling to see the content.”

(FGD4, Male)

### Scalability

Students expressed strong support for expanding the use of eSHEPP, emphasizing the importance of collaboration among teachers, parents, and schools to maintain and extend the program’s impact. They suggested that integrating eSHEPP into the regular school curriculum could encourage sustained participation and promote health awareness across the wider community.

“Implementing this program in every school would benefit all students equally.”

(FGD4, Male)

“The program should expand, more activities and more participants, so everyone can benefit from this learning experience.”

(FGD1, Female)

Participants also highlighted that shared responsibility between teachers and parents would enhance the program’s reach and effectiveness. Teachers were seen as key facilitators who could incorporate eSHEPP content into existing lessons, while parental involvement was considered crucial for reinforcing learning at home.

“Teachers can amplify this program’s reach, they already have organized classes where they can share the content.”

(FGD1, Female)

“Parents should receive this too; they’ll share it with the whole family, making the lessons reach more people.”

(FGD3, Male)

In addition, students noted that including eSHEPP as part of the school curriculum would support long-term sustainability and motivate learners to engage seriously with the sessions.

“Integrating this program into the school curriculum would give it the importance it deserves, ensuring all students engage seriously.”

(FGD2, Female)

**Figure 3:**
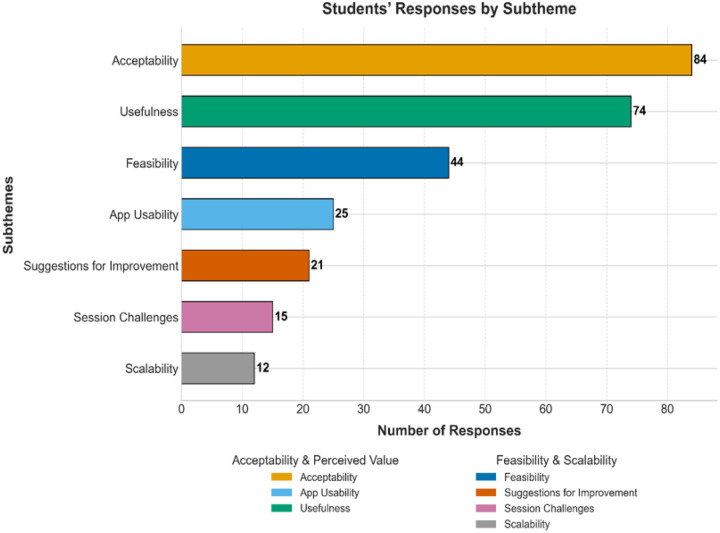
Distribution of students’ responses by subtheme within two overarching themes: Acceptability and perceived value of a narrative-based eHealth intervention and Feasibility and scalability within school systems.

Figure 3 shows how student responses were distributed across subthemes within two main areas: Acceptability and Perceived Value of a Narrative-Based eHealth Intervention and Feasibility and Scalability within School Systems. While our analysis focuses on understanding themes in depth, the frequencies here help illustrate which topics were most prominent in student discussions, without implying any statistical conclusions (39). Responses were most frequent for subthemes under Acceptability and Usefulness, especially Overall Acceptability and Knowledge Improvement, suggesting strong engagement and perceived benefit. The full distribution of responses across all subthemes and categories can be found in Supplementary Material 2.

## DISCUSSION

### Principal Findings

Secondary and higher secondary school students in Karachi responded positively to eSHEPP, describing the intervention as acceptable, feasible, and beneficial. Two overarching themes emerged: high acceptability and perceived value, and strong feasibility with potential for scalability within school systems. Together, these themes reflect the close alignment of the intervention with students’ needs, preferences, and contextual realities.

Multimedia video sessions, featuring concise dramatizations, clear language, and supportive subtitles, were consistently described as engaging and easy to follow. Students reported applying the content in their daily lives, such as adopting healthier eating habits, increasing awareness of physical activity, and discouraging smoking among peers, suggesting behavior change that extended beyond the classroom. These findings align strongly with the Technology Acceptance Model, as students emphasized both perceived usefulness (knowledge gained and lifestyle modifications) and ease of use (clarity of the videos and overall app usability). The Technology–Task Fit model was also supported, with the program fitting well within students’ academic schedules, available school resources, and preferred learning styles, enabling seamless integration without disrupting routine activities.

The qualitative findings were organized into two overarching themes with their corresponding subthemes rather than a larger set of independent themes. This structure reflected the focused and theory-driven nature of the dataset, in which participants’ responses aligned closely with the constructs of the TAM and TTF framework. Using two overarching themes allowed for greater conceptual clarity and analytical depth, ensuring coherence between the theoretical framework and the emergent data. This approach avoided unnecessary fragmentation of closely related insights and accurately represented students’ perceptions of eSHEPP’s acceptability, usefulness, feasibility, and scalability.

### Comparison With Prior Work

Our findings offer rich and in-depth insights into adolescents’ experiences with eSHEPP, particularly regarding acceptability, perceived usefulness, engagement, and feasibility. Participants emphasized that interactive content, relatable dramatized narratives, and a user-friendly design enhanced their motivation, comprehension, and overall perceived value of the program (40–44). For example, adolescents participating in smartphone-based oral health and mental health interventions have highlighted the importance of clear instructions, interactivity, and opportunities for discussion (40–42). Other qualitative studies have reported that access to culturally relevant and personalized digital tools, along with careful attention to privacy and localization, are key factors influencing adolescents’ motivation and sustained engagement with digital health programs (43, 44).

The findings also showed that adolescents’ engagement and perceived benefits were shaped by the intervention’s usability, its relevance to the school context, and its practical applicability to daily life. Many students reported influencing their peers and family members, which is consistent with previous qualitative evidence from digital storytelling and mHealth interventions that highlights adolescents as agents of change within their communities (40–44). Participatory design studies further indicate that aligning digital programs with school schedules, available resources, and local learning contexts enhances both feasibility and scalability (41, 45–47).

Beyond qualitative comparisons, our findings are supported by broader quantitative and systematic evidence. Systematic reviews and meta-analyses indicate that digital interventions that incorporate peer or teacher support can improve adolescents’ knowledge, health behaviors, and overall well-being (48–51). Program evaluations, including edutainment-style video interventions in Thailand (RCT) (52), a YouTube Health video in Germany (53), LIFE4YOUth in Sweden, and an mHealth lifestyle app in Spain (54, 55), have demonstrated measurable improvements in health knowledge and lifestyle behaviors in both school and community settings. The influence adolescents reported having on peers and family members in eSHEPP aligns with previous evidence from digital storytelling and mHealth programs that promote community-level behavior change (56, 57). Collectively, these studies provide contextual support for our findings and highlight that narrative-driven, interactive digital interventions are feasible and effective across diverse adolescent populations.

Overall, by integrating qualitative and quantitative evidence, this work underscores the importance of interactive, contextually relevant, and narrative-driven design in school-based digital health interventions. It also contributes context-specific insights from Pakistani adolescents, extending the existing evidence base and highlighting considerations for implementation in similar settings.

### Study Contributions and Novelty

To our knowledge, this is the first qualitative study in Pakistan to explore the acceptability and feasibility of a school-based digital health education program for adolescents. By focusing on these key areas, the study provides insights into how narrative-based, app-supported health education can be implemented effectively in low-resource school settings. The findings are intended to inform integration into Pakistan’s National Health Vision (2016–2025) and Digital Pakistan Policy (2018) and to guide the sustainable scale-up of digital adolescent health interventions in schools (58, 59).

The eSHEPP app played a central role in these positive outcomes. As a blended digital learning platform, it combined dramatized storytelling, short educational videos, and interactive quizzes to reinforce key messages. Its Urdu-language interface, simple navigation, and multimedia elements made learning accessible and engaging for students from diverse backgrounds. Beyond delivering information, the app encouraged self-paced review, reflection, and classroom discussion, supporting deeper understanding and retention of health concepts. The combination of interactive and narrative-based design elements likely strengthened the program’s impact on knowledge, attitudes, and behaviors, demonstrating how a well-designed digital platform can facilitate learning and behavior change in adolescent health promotion.

### Strengths and Limitations

A key strength of eSHEPP is its ability to deliver health education without disrupting academic routines. The program requires only basic equipment and combines app-based learning with in-person facilitation. Students also provided valuable suggestions for improvement, including expanding health topics, slightly lengthening sessions, and adding interactive app features such as games, voice recording, or peer chat. These recommendations offer clear opportunities to enhance the program further. Although the study focused specifically on user experience and delivery feasibility, this targeted approach enabled rich, actionable insights that can inform future program adaptation and scaling.

Several limitations should be noted. The study relied on self-reported focus group data, which may be influenced by social desirability bias, and it did not include objective measures of behavior change. Future research could strengthen validity by integrating qualitative insights with objective indicators, such as physical activity logs, step counts, or dietary recalls. Evaluating the program in rural and lower-income schools would also help assess its generalizability and scalability across diverse contexts.

### Future Directions

Given the strong feasibility and scalability demonstrated in this pilot, expanding eSHEPP to additional schools and involving families could increase its reach. Integrating the program into formal curricula, supported by active teacher facilitation and parental reinforcement, may help sustain long-term benefits. This approach aligns with frameworks for sustainable, low-barrier adolescent health promotion (51).

Specifically, aligning eSHEPP with Pakistan’s National School Health Policy and the WHO Global Standards for Health-Promoting Schools could support institutionalization and long-term adoption. Achieving sustainability will require system-level integration and policy support. Evidence from other low- and middle-income countries suggests that embedding digital health education into national curricula, with government and donor backing, can enhance both reach and effectiveness (60). Ensuring equitable access remains essential, particularly for students without smartphones or optimal classroom environments. Future studies should adopt mixed-method and longitudinal designs, pilot the program in underserved areas, and leverage real-time app analytics to monitor engagement and adapt content dynamically.

## CONCLUSION

eSHEPP was found to be a highly acceptable, feasible, and user-friendly approach for delivering adolescent health education in low-resource school settings. Its multimedia, app-based format engaged students, enhanced their understanding of health topics, and promoted positive behaviors that extended beyond the classroom, influencing peers and family members.

Strengthening eSHEPP’s content, interactivity, and delivery, alongside integration into school curricula with teacher and parental support, positions it as a promising, scalable, and sustainable model for adolescent health promotion. Future research employing robust, mixed-method, and longitudinal designs should evaluate its effectiveness and long-term impact across diverse educational and community contexts.

## Supplementary Material

Supplementary Files

This is a list of supplementary files associated with this preprint. Click to download.

- SupplementaryMaterial123.docx
- SupplementaryMaterial4COREQchecklist.docx
- SupplementaryMaterial5Codebook.docx
- SupplementaryMaterial6Focusgroupdiscussionguide.docx

## Data Availability

The deidentified interview transcripts analyzed during the current study are not publicly available due to confidentiality concerns but may be made available from the corresponding author upon reasonable request and subject to approval from the ethics committee.

## Implications for Policy and Practice

- School-based digital health education is feasible in low-resource settings, requiring minimal equipment and not disrupting academic routines.
- Multimedia, app-supported learning effectively engages adolescents; dramatized videos combined with guided discussions enhance knowledge retention, promote behavior change, and generate spillover effects among families and peers.
- Curriculum integration and stakeholder engagement are critical; embedding eSHEPP into formal school curricula with active teacher facilitation and parental reinforcement can support sustainability and long-term impact.
- Policy and donor support can expand the reach and sustainability of adolescent digital health programs, helping address the growing burden of noncommunicable diseases among youth in low-and middle-income countries.

## Abbreviations

COREQ: Consolidated Criteria for Reporting Qualitative Research
FGD: Focus Group Discussion
LMICs: Low- and Middle-Income Countries
NCDs: Noncommunicable Diseases
eSHEPP: School eHealth Education Program Pakistan
TTF: Task–Technology Fit
TAM: Technology Acceptance Model
WHO: World Health Organization
